# Consumers' Impulsive Buying Behavior in Online Shopping Based on the Influence of Social Presence

**DOI:** 10.1155/2022/6794729

**Published:** 2022-07-01

**Authors:** Mingming Zhang, Guicheng Shi

**Affiliations:** School of Business, Macau University of Science and Technology, Macau 999078, China

## Abstract

The rapid development of online shopping has contributed to marketing strategy. Social presence plays an important role in the field of marketing. Therefore, this paper studies the influence of social presence on online impulse buying. The key marketing strategy is that consumers make impulsive buying behavior in online shopping. This paper proposes a research scheme for an impulsive buying sharing model based on user features and an article studied before which has some factors presented. The model is evaluated on a data analysis based on SPSS24.0 and AMOS23.0. The results show that the main factors such as interactivity, vividness, and media richness, all have positive effects on social presence. Therefore, in the variable relationship, social presence has a direct impact on impulsive buying behavior. This result has a theory contribution on the marketing theory model which also has an important practice significance in marketing strategy for enterprises.

## 1. Introduction

With the development of online shopping, consumers' shopping methods can be divided into rational purchases and impulsive purchases. Therefore, it is necessary to study impulsive shopping behavior for which social presence is the key factor in online shopping. Gunawarden [1] defined social presence as the perception formed by participants during their online participation, which emphasizes satisfaction or the real perception of others in video conferencing interactions. Research studies show that social presence is a strong predictor of satisfaction and that participants with strong social presence use emoticons to express nonverbal cues to enhance their social emotional experience. It has also been found that the interactive nature of websites can promote the comparison of personal presence and that the social presence conveyed by websites can influence behavior intention by influencing one's enjoyment and perceived usefulness. It has also been found that social presence can improve consumers' safety perception and purchase attitudes in regard to virtual shopping. However, there is no empirical study on the direct impact of social presence on impulsive buying. Based on the current research on impulsive buying in online shopping, this paper mainly discusses the main antecedents of social presence in online shopping, discusses how these factors affect social presence, and then discusses how social presence in online shopping affects impulsive buying behavior. Finally, the current paper discusses how customers' impulsive buying tendency impacts their impulsive buying behavior in online shopping. This paper is organized as follows: Section 1 discusses what are the main variables of social presence in online shopping.  Section 2 is about how about these factors affect the social presence.  Section 3 discusses how social presence affects impulse buying in online shopping.  Section 4 explores how did customers' impulsive buying tendency modulate consumer on impulsive buying behavior.

## 2. Related Work

### 2.1. Impulse Buying Behavior

Rook and Fisher [2] defined impulsive buying behavior as the description of the thoughts and emotions experienced by consumers in the case of impulsive buying. This is a kind of purchase behavior that is not controlled by emotion. Many scholars have studied and defined impulsive purchase behavior.

Piron [3] defined impulsive buying according to three aspects, namely, an unplanned purchase, emotional stimulation, and the timeliness of the behavior, which is seen as the purchase behavior of making decisions immediately; the author also divided impulsive buying behavior into experiential impulsive buying and nonexperiential impulsive buying. However, later studies further pointed out that impulsive purchases were accompanied by emotional reactions. Wood [4] believed that impulsive purchase was an unplanned purchase without careful consideration and accompanied by high emotional conflicts. Parboteeah et al. [5] showed that external environmental stimulation may significantly affect consumers' perception of product usefulness and hedonism, thus affecting their impulsive purchase intention. It can be seen that the stronger the consumers' knowledge of the usefulness, the more positive mood in buying intention, thus further promoting impulsive buying.

### 2.2. Social Presence in Online Shopping

The idea of social presence originated from the two concepts of directness and intimacy, which were involved in the study of interpersonal interaction in the field of social psychology and mainly focus on remote exchange media by Cui et al. [6]. Gefen and Straub [7] introduced the concept of social presence into the field of consumption research and found that the level of customers' trust in electronic products and services was strongly affected by social presence. However, in the context of online live shopping, the “human-to-machine contact experience” was completely different from the social presence brought about by the “human-to-human contact experience” on the live platform.

In the online broadcasting room, consumers can see the physical display of sellers on the other side of the communication port in real time with the help of the live broadcasting platform, and they can communicate with the sellers through text. Generally, the live broadcast interface is attached to a payment link through which one can place an order directly and these transactions can be concluded directly under the guidance of the seller. This means that given the real-time physical display and the seller's hard sales guidance, consumers can make consumption decisions quickly.

### 2.3. Interactivity

Interactivity has been defined in many ways. For example, Blattberg and Deighton [8] defined interactivity as the facility for persons and organizations to communicate with another regardless of distance or time directly. Steuer [9] suggested that interactivity was “the extent to which users can participate in modifying the format and content of a mediated environment in real time.” Nowak et al. [10] defined interactivity as the degree of interaction through media and machines. From the perspective of consumers, the communication between consumers and sellers through the live broadcasting platform was bidirectional and synchronous [11, 12]. Because of its unique feature as a medium that was not only received but also transmitted, when people have positive interactivity, the social presence was shown in the live broadcasting room.

### 2.4. Vividness

Vividness was the degree of connected objects that combine the sensory experience of actual objects with the nonsensory experience of hallucinations [13]. In the context of e-commerce, vividness has been widely used in product quality demonstrations [14]. Similar to interactivity, vividness also help consumers imagine and experience products in a future consumption environment [15]. Online shopping platforms use colors, charts, music, and video to stimulate consumer's visual, auditory, and other senses [16]; however, these were relatively static in traditional online platforms. The live shopping platform can provide an all-around display and comparison in real time.

Both interactivity and vividness significantly affected social presence and indirectly affect participation. Engagement has the most significant effect on all advertising effectiveness indicators, i.e., greater than the initial hypothesis of this study. The key role of engagement found the process of buying consumers may interact with new media; it is a more conservative emotional process that is more consistent with the information processing view than initially thought, thereby supporting the usage and satisfaction paradigm [17]. If, from a cognitive perspective, the media itself is more participatory than other media, then the increase in engagement of a particular advertisement within that media may depend more on emotion than on the cognitive dimension.

### 2.5. Media Richness

Media richness, as proposed by Daft and Lengel [18], refers to the media's capacity to facilitate shared meaning and understanding. The communication environment created by enterprises and the diversification of communication means are the key premises of creating media diversity. The live broadcast platform not only provides interaction between the anchor and consumers but also arranges special online customer services in the sales link to assist customers in placing orders and engaging in after-sales communication. They argued that organizations process information to reduce levels of uncertainty (lack of information) and ambiguity (multiplicity). They also argued that this task improves the ability to convey information when the task needs to be matched with the media, a better use of the media to convey “rich” information improves the task. It is also believed that the richness of the website and its more social, nonverbal, and complex cues, all contribute to consumers having a positive attitude towards the website. Thus, a rich online shopping environment has a positive impact on consumers' online purchase intention. This result was not surprising, as previous studies have found that consumers prefer rich looks, regardless of the nature of the product. Consumer attitude is the strongest factor influencing online shopping intention by Mattila [19].

Therefore, we summarize the existing literature on social presence and e-commerce, combined with the interactive characteristics of live broadcasting platforms; this study argued that interactivity, vividness, and media richness mainly affect social presence. Interactivity was considered to be a kind of environment which users can participate in and make adjustments to from time to time [9].

### 2.6. Moderator Variable

Moderating variables can be qualitative (such as gender, race, school type, etc.) or quantitative (such as age, years of education, number of stimuli, etc.), and they affect the direction (positive or negative) and strength of the relationship between the variable and the number of arguments. The research history and application of impulsive buying traits as a moderating variable will be reviewed in the following. The moderating effect of impulsive buying tendency.

Wu and Guo [20] took individual impulse buying traits as a moderating variable, and their research results showed that individual impulse buying traits played a moderating role in the relationship between flow experience and impulse buying, as well as in the relationship between website interaction and vividness through flow experience and impulse buying. Jones et al. [21] believed that impulsive buying tendency was defined as the buying process of individuals that have no thinking and unplanned which is impulsive buying behavior. This trait can predict the likelihood of impulsive purchase. Therefore, in the field of marketing and consumer behavior research, many scholars have done a lot of research on impulsive purchase tendency. Different online consumers have different personality traits, which affects consumers' willingness to shop in online stores and their final behavioral decisions. Studies have proven that consumers' personality traits have a moderating effect on situational factors and purchasing.

## 3. Hypothesis Development

### 3.1. Interactivity and Social Presence

The existing literature points out that interactivity is a key factor affecting social presence by Hassanein and Head [22]. Research studies on the impact of website presence show that vividness and interactivity are effective means by which to manipulate the level of presence [17]. Therefore, this paper believes that interactivity will have a positive impact on social presence. This study proposes the following research hypothesis:

H1: Interactivity has a positive impact on social presence in online shopping.

### 3.2. Vividness and Social Presence

Vividness can improve depth through richness, which refers to the width and breadth of the quality of information perceived by media users and to the sensory dimension that a communication medium can provide (Daugherty et al. [16]). The more vivid the performance of the interactive process of online shopping is, the stronger the telepresence effect is, the higher the trust level of consumers is, and the stronger the purchase intention is. Liu et al. [23] and others have confirmed that visual attraction can significantly improve immediate satisfaction and thus affect the display of impulse purchase goods. It is more comprehensive and multiangle way to present information to consumers through a live screen than in the pictures that are used in traditional online shopping. Video displays are also more comprehensive. According to the theory of multisensory interaction and integration, a good visibility effect can increase the level of virtual touch and enhance the presence of online shopping. Therefore, this study puts forward the following research hypothesis:

H2: Vividness has a positive impact on social presence in online shopping.

### 3.3. Media Richness and Social Presence

Parker et al. [24] put forward the concept of social telepresence. They called the degree of people's perception of the significance of others and the presentation of this interpersonal relationship through the media via the process of using the media for communication “social telepresence”, which has the ability to convey nonverbal clues and prompt the social context when there is a higher level of social presence compared to when the degree of social presence is low. The media enrichment theory came into being under this academic background. Although media enrichment theory and social telepresence theory have different definitions of media attributes, they both follow a similar position; i.e., media selection depends on the characteristics of the media, and each communication media has a unique ability to convey some informational content, which has an important impact on the appropriateness of media selection. As an open social system, an organization needs to continuously reduce the “difference between the amount of information required to perform tasks and the amount of information already owned by the organization” by Galbraith and Underwood [25]. Thus, the amount of information is a key factor in an organization's information-processing activities. Daft and Lengel [18] interpreted the concept of information richness as “the ability of information to change understanding and cognition over a period of time”. Some scholars have found that media richness affects social telepresence. The social telepresence theory believes that media can affect social telepresence, which is also related to the media richness theory. A high degree of social telepresence is typical in face-to-face communication. Therefore, this study puts forward the following research hypothesis:

H3: Media richness has a positive impact on social presence in online shopping.

### 3.4. Social Presence and Online Impulsive Buying Behaviour

Waller and Bachmann [26] point out that because trust arises in the social environment, social presence is a necessary condition for generating trust. An environment with a rich social presence can stimulate a sense of trust among participants. Song et al. [27] found that the stronger the presence of consumers on the clothing sales website is, the higher the trust level of online consumers is. Hausman and Siekpe [28] found that intoxication has a significant positive impact on consumers' online revisit intention and purchase intention. Korzaan and Boswell [29] also found that consumers' intoxication during browsing websites helps to improve their consumption attitude and purchase intention. Animesh et al. [30] found a study on 3D virtual space that social presence can promote users' purchase intention. Therefore, this study puts forward the following research hypothesis:

H4: Social presence has a positive impact on online impulsive buying behavior.

## 4. Research Methodology

### 4.1. Study Location and Sample

The data collection for the current study was greatly supported by an electronic questionnaire, which was divided into two stages. One stage was based on the principle of judgment sampling surveys and was presented in the form of a questionnaire that was sent through WeChat, electronic mail, and other social media platforms, as well as forwarded for use through the network broadcast platform, and was mainly distributed in Guangdong, Macau, Beijing, Shandong, Northeast China, and other places for volunteers to fill out and submit. The questionnaire used in this study was mainly distributed through Internet research, and the survey subjects were people who had impulsively consumed goods in an online live broadcast room. The surveyed was completed mainly by social people and was supplemented by graduate and undergraduate students.

Initially, 340 self-questionnaires were distributed during a year of field visits and survey collection. After deleting incomplete and mismatched questionnaires, 319 valid questionnaires (93.8%) were retained and ultimately constituted the research sample. Among the respondents, 63.23% were female and 36.76% were male. Regarding the educational level, 3.82% of the respondents had finished middle school or below, 8.24% had finished junior college, 44.41% had finished a bachelor's degree, 31.47% held a master's degree, and 12.06% held a doctoral degree. The ages of the online shopping consumers were as follows: 3.82% were under the age of 20; 37.06% were between 21 and 30; 37.35% were between 31 and 40; 13.53% were between 41 and 50; and 8.23% were above 50. The annual incomes of the respondents were as follows: 21.76% had an annual income below RMB 150,000 (RMB 1,000 = approximately USD 140); 24.12% had an annual income between RMB 160,000 and 300,000; 20.59% had an annual income between RMB 310,000 and 450,000; 10.59% had an annual income between RMB 460,000 and 600,000; and 22.94% had an annual income above RMB 610,000.

### 4.2. Measures

All the scales used in this study are mature scales that have been used in international journals which have good reliability and validity. Descriptive statistics is conducted for the items located in the questionnaire survey of this study, mainly including average values and the standard differences of the variables, and the data distribution states of the measurement items were judged according to each value.

### 4.3. Reliability Analysis

First, the reliability of each variable dimension measurement index was determined. Reliability mainly tests the consistency and reliability of variables in a study for each measurement item to ensure the validity of the model fitting degree. Nunnally (1998) believed that an alpha coefficient greater than 0.9 indicates the best reliability, a value between 0.7 and 0.9 indicates good reliability, a value between 0.35 and 0.7 indicates medium reliability, and a value less than 0.35 indicates low reliability. As Devellis (1991) suggested, a Cronbach's alpha value greater than 0.7 indicates that the reliability is satisfactory. Therefore, reliability is a method by which to measure internal consistency. The higher the coefficient is, the higher the consistency and reliability of the data are.

As seen from Table 1, the alpha coefficients of the prevariables of interactivity, vividness, and media richness in this study are 0.925, 0.855, and 0.890, respectively. The alpha coefficient of the core variable of social presence is 0.935. The alpha coefficient of impulsive buying behavior is 0.878. The alpha coefficient of the moderating variable impulse buying tendency is 0.896. Cronbach's alpha coefficient reference indices are all larger than 0.7, which indicates that each variable in the study has good reliability.

As seen from Table 2, the main purpose of confirmatory factor analysis (CFA) is to validate validity and to analyze common method bias (CMB). There are many kinds of validity, such as content validity, structure validity, convergence validity, and discriminant validity. The differences in each type are described as follows. As seen from the above table, a total of 7 factors and 48 analysis items were analyzed by CFA. The effective sample size of this analysis was 340, which was 5 times more than the number of analysis items but less than 10 times the number of analysis items.

As seen from Table 3, the AVE values corresponding to the total of 7 factors are all greater than 0.5, and the CR values are all higher than 0.7, which means that the analyzed data have good convergence (convergence) validity. At the same time, the corresponding factor loading value of each measurement item is generally required to be greater than 0.7. Sometimes, model fitting indices and model MI values may be combined to achieve better conclusions.

### 4.4. Correlation Analysis between Variables

As seen from Table 4, the correlation value between PP and IBB was 0.222 and showed a significant level of 0.01, indicating that there was a significant positive correlation between PP and IBB. The correlation value between PP and IBT was 0.116, and it was significant at the level of 0.05, indicating that there was a significant positive correlation between PP and IBT. Correlation analysis was used to study the correlations between PP, IBB, and IBT, and the Pearson correlation coefficient was used to indicate the strength of these correlations. The correlation value between PP and IBB was 0.222 at a significance level of 0.01, which indicates a significant positive correlation between PP and IBB. The correlation value between PP and IBT was 0.116 at a significance level of 0.05, which indicates a significant positive correlation between PP and IBT.

### 4.5. Structural Equation Model

Structural equation modeling (SEM) is a confirmatory analysis method that is used for multivariate statistical analysis. Through the reliability and validity testing of the questionnaire, it was concluded that all the scales used in this study have good reliability and validity. Furthermore, AMOS 23.0 was used for hypothesis testing to verify whether the model fitting degree and path were significant and to test whether the independent variable had a significant impact on the dependent variable. AMOS uses the chi-square value as the fitting test result, and the fitting degree usually uses CMIN/DF, GFI, RMR RMSEA, AGFI, NFI, CFI and IFI as indicators.

#### 4.5.1. Structural Equation Model Testing of Interactivity, Vividness, MR, and SP

As seen from Table 5, the SEM regression relation table of a structural equation model includes two kinds of relations, namely, the influence structure relation and the measurement relation. The normalized path coefficient value is usually used to represent the relationship, i.e., whether it affects the structure relationship or the measurement relationship. If this value is significant, this indicates that there is a significant influence/measurement relationship; otherwise, there is no influence/measurement relationship between the description items. If more path coefficients are not significant compared to those that are significant, this indicates that the model is poor. In this case, it is recommended to reset the model relationship, that is, to adjust the model. In this study, *p* values between the interactivity, vividness, and MR and SP variables were all less than 0.05. Interactivity had a significant positive effect on social presence (*β* = 0.205, *p* < 0.001), and vividness had a significant positive effect on social presence (*β* = 0.43, *p* < 0.001). Finally, media richness has a significant positive impact on social presence (*β* = 0.208, *p* < 0.001), and the hypothesis is valid, which indicated that these three variables all had positive and significant effects on SP; thus, our hypothesis was valid. Social presence has a significant positive impact on impulsive buying behavior (*β* = 0.037, *p* < 0.001) and the hypothesis is true. The hypothesis holds that price promotion has a significant positive effect on impulse buying behavior (*β* = 0.162, *p* < 0.001).

#### 4.5.2. Test of Model Fitting Degree

As seen from Table 6, the results of the software output were used to verify the fit of the model. The model suitability test indicate involved in the SEM included 10 categories and up to 38 index test quantities. There are three commonly used fitness test indicates, namely, absolute fitting indicate, relative fitting indicate, and simplified fitting indicate. The meanings and discriminant criteria of each index are as follows.

#### 4.5.3. Goodness-of-Fit Index (GFI)

Since the goodness of a chi-square test depends on the sample size, the goodness-of-fit index (GFI) does not depend on sample size for easy measurement; rather, the goodness-of-fit index can measure the degree to which the covariance matrix predicts the S matrix. When the GFI is 1, it is an ideal model; however, during modeling and analysis, it is generally believed that if the GFI value is greater than 0.9, the model results are ideal. In this study, the GFI was 0.893, which is close to 0.9. Thus, it can be judged that the model fits well.

#### 4.5.4. Modified Goodness-of-Fit Index (AGFI)

The AGFI is similar to the GFI in that the closer it is to 1, the better the fitting effect is. Generally, the value of the AGFI is required to be at least greater than 0.85; however, for the same model, the AGFI must not be greater than the GFI. In this study, the AGFI was 0.877, which is greater than 0.85, and it was less than the GFI; thus, the model fitting effect was good.

#### 4.5.5. Root Mean Square of Approximate Error (RMSEA)

Among the numerous fitting indicates, the probability of making type I and type II errors is small, which presents a reasonable fitting index. The closer the fitting index is to 0, the better the fitting effect of the model. In this study, the root mean square error value was 0.034, which is lower than 0.1; this indicates that the model had a good fitting effect.

#### 4.5.6. Akaike's Information Criterion, AIC

AIC is often used to evaluate general statistical models. The better the model fits, the smaller the AIC value will be; however, the value is affected by the number of samples and parameters in the model. A large number of samples and parameters will easily cause a bad model to be mistaken for a good one.

#### 4.5.7. Standardized Root Mean Square Residual (SRMR)

For SRMR, some models are not greatly affected by N, while others are greatly affected by N. Hu and Bentler believed that when SRMR < 0.8, the model fitting effect is better. In this paper, the SRMR value was 0.036, which is less than 0.8. Therefore, it can be considered that the overall fitting effect of the model was good.

## 5. Discussion

In this paper, the structural equation model tested by data collection and investigation, and the correlations between them were discussed. Through research methods such as theoretical deduction, model, and statistical tests, which were used to determine the main characteristics of compulsive purchase behavior in regard to online shopping variables, the main variables affecting social presence, impulsive purchase, and consumer personality tendencies, with core variables focusing on the relation between social presence and action, formed the following four conclusions.

First, the literature review shows that with the rapid development of the Internet, online shopping is more convenient than offline shopping for many platform enterprises. At present, platform enterprises are still developing operation plans for offline brands; therefore, it is very important for enterprises to study the impulsive buying behavior of consumers in relation to online shopping.

Second, the influence of interactivity, vividness, and media richness on social presence and the corresponding influence path have been clarified. The empirical results show that interactivity, vividness, and media richness, all have positive effects on social presence. Therefore, in online shopping, the atmosphere felt by consumers in the live broadcast room and the characteristics presented in the live broadcast room must meet the premises of interaction, vividness, and media richness in order to have a positive impact on consumers' impulse buying behavior.

Third, the current study proves that social presence has a direct influence on impulsive buying behavior through the variable relationship. This outcome shows that in the context of Internet shopping, the sense of social presence is a core variable, and consumers are prone to impulsive buying behavior when they perceive that they are physically present, i.e., if they feel that they are really in the live broadcast room. Social presence plays an important role, and the user's experience of social presence directly affects his or her degree of impulse consumption. Therefore, the challenge of how to make consumers have a sense of social presence is a core issue when constructing online live shopping experiences.

Fourth, the current study verifies the positive moderating effect of consumers' personal trait of having an impulse buying tendency on social presence and impulse buying behavior. Among these factors, the higher the impulse buying tendency is, the stronger the positive impact of social presence on impulse buying behavior is. The research study of foreign scholars has also confirmed that impulse buying tendencies have a positive response on impulse buying behavior.

## 6. Conclusion

An effective marketing module is proposed in this paper, which is based on the SOR theory module and predecessors research, aiming at the problem of online impulsive buying. In order to verify, the module is added to the feature extraction network. Experiments were carried out on the data analysis, the module gets the best performance in the data analysis index GFI gets 0.903, and the results show that the module proposed in this paper can improve the hypothesis.

The innovation of this paper are two theoretical contributions. First, the concept of social presence comes from the field of psychology. The concept has attracted attention from brand management, marketing, and communication studies. Second, the academic contributions of this study mainly include the following two aspects: the variables affect the social presence of platforms and establishing the relevant of impulsive buying behaviors. Previous research on social presence has mainly focused on artificial intelligence, online education, and VR, which has filled the gap in brand affection research on live broadcast platforms and provided new ideas for follow-up research on brand affection in the Internet content.

### 6.1. Limitations and Further Research

For the factors related to impulsive buying behavior in online shopping, this paper only focuses on the influence of the three leading variables of interactivity, vividness, and media richness; it does not consider the mediating or moderating effects of other variables in the influencing path. Undeniably, the relevant factors affecting impulsive buying behavior in online shopping are complex and diverse; there is even a model of other unknown factors that influence impulsive buying behavior in online shopping in the Internet context. It is hoped that further research can further explore more potential influencing factors, intermediary variables, and moderating variables in order to supplement and improve the results of this study and make further contributions to the study of online shopping platforms. There is a lack of discussion on social presence in the field of live broadcasting. Social presence is a multidimensional concept, and the degree of its strength differs in different product categories or industries. Therefore, the influence of various dimensions of social currency on brand affection can be studied on this basis in the future. Therefore, research on consumers' impulsive buying behavior in relation to online shopping requires more in-depth and comprehensive discussion.

## Figures and Tables

**Table 1:** The Cronbach's reliability analysis.

Name	(CITC)□	Alpha *α*□	Cronbach *α*□
SP1	0.745	0.928	0.935
SP2	0.691	0.931	
SP3	0.699	0.93	
SP4	0.711	0.929	
SP5	0.781	0.926	
SP6	0.764	0.927	
SP7	0.728	0.929	
SP8	0.79	0.926	
SP9	0.735	0.928	
SP10	0.776	0.926	

Cronbach *α*: 0.936□			
Inter1	0.676	0.919	0.925
Inter2	0.648	0.92	
Inter3	0.635	0.921	
Inter4	0.626	0.921	
Inter5	0.753	0.915	
Inter6	0.75	0.915	
Inter7	0.684	0.918	
Inter8	0.76	0.915	
Inter9	0.645	0.92	
Inter10	0.763	0.915	
Inter11	0.762	0.915	

Cronbach *α*: 0.926□			
Vivid1	0.7	0.818	0.855
Vivid2	0.618	0.843	
Vivid3	0.737	0.81	
Vivid4	0.595	0.845	
Vivid5	0.722	0.813	

Cronbach *α*: 0.859□			
MR1	0.667	0.876	0.89
MR2	0.574	0.884	
MR3	0.69	0.874	
MR4	0.707	0.872	
MR5	0.676	0.875	
MR6	0.653	0.877	
MR7	0.724	0.87	
MR8	0.641	0.878	

Cronbach *α*: 0.891□			
IBT1	0.748	0.874	0.896
IBT2	0.658	0.892	
IBT3	0.771	0.868	
IBT4	0.772	0.868	
IBT5	0.785	0.864	

Cronbach *α*: 0.898□			
PP1	0.779	0.883	0.906
PP2	0.788	0.879	
PP3	0.807	0.873	
PP4	0.783	0.881	

Cronbach *α*: 0.907□			
IBB1	0.684	0.859	0.878
IBB2	0.643	0.869	
IBB3	0.693	0.857	
IBB4	0.801	0.833	
IBB5	0.747	0.844	

Cronbach *α*: 0.881□			

**Table 2:** CFA analysis.

Factor	Amounts
SP	10
Inter	11
Vivid	5
MR	8
IBT	5
PP	4
IBB	5
All	48
Sample amounts	340

**Table 3:** The results of convergence validity analysis.

Factor	AVE	CR
SP	0.59	0.935
Inter	0.53	0.925
Vivid	0.542	0.855
MR	0.507	0.891
IBT	0.638	0.897
PP	0.708	0.907
IBB	0.593	0.879

**Table 4:** Correlation analysis.

	Mean	Standard deviation	P	IB	IB
PP	2.45	0.77	1		
IBB	2.961	0.782	0.222^*∗∗*^	1	
IBT	2.893	0.771	0.116^*∗*^	0.189^*∗∗*^	1

^*∗*^ *p* < 0.05,^*∗∗*^*p* < 0.01.

**Table 5:** Structural equation model coefficient parameter table.

Research route			Standardized coefficient	Nonstandardized coefficient	S.E.	C.R.	*p*
SP	<---	Inter	0.204	0.272	0.074	3.675	^*∗*^ ^*∗*^ ^*∗*^
SP	<---	Vivid	0.427	0.588	0.093	6.344	^*∗*^ ^*∗*^ ^*∗*^
SP	<---	MR	0.217	0.293	0.077	3.816	^*∗*^ ^*∗*^ ^*∗*^
IBB	<---	SP	0.344	0.337	0.06	5.642	^*∗*^ ^*∗*^ ^*∗*^
PP	<---	IBB	0.174	0.162	0.054	3.011	0.003

**Table 6:** Model fitting index.

Item	*χ* ^2^	df	*p*	*χ* ^2^/df	GFI	RMSEA	RMR	CFI	NFI	NNFI
Standard	—	—	>0.05	<3	>0.9	<0.10	<0.05	>0.9	>0.9	>0.9
Value	727.461	521	0	1.396	0.893	0.034	0.024	0.969	0.899	0.966
Others	TLI	AGFI	IFI	PGFI	PNFI	SRMR	RMSEA 90% CI			
Standard	>0.9	>0.9	>0.9	>0.9	>0.9	<0.1	—			
Value	0.966	0.877	0.969	0.782	0.834	0.036	0.028∼0.040			

Default Model: *χ*^2^(561) = 7168.007, *p* = 1.000.

## Data Availability

The data used to support the findings of this study are available on request from the corresponding author.
